# Caring for depression in the dying is complex and challenging – survey of palliative physicians

**DOI:** 10.1186/s12904-022-00901-y

**Published:** 2022-01-16

**Authors:** Wei Lee, Sungwon Chang, Michelle DiGiacomo, Brian Draper, Meera R. Agar, David C. Currow

**Affiliations:** 1 University of Technology Sydney, Improving Palliative, Aged and Chronic Care through Clinical Research and Translation (IMPACCT), Ultimo, NSW 2007 Australia; 2 St Vincent’s Clinical School, University of New South Wales, 390 Victoria St, Darlinghurst, NSW 2010 Australia; 3 School of Psychiatry, University of New South Wales Sydney, Sydney, NSW 2052 Australia; 4 Faculty of Science, Medicine and Health, University of Wollongong, Wollongong, NSW 2522 Australia

**Keywords:** Depression, Palliative care, Psychiatry, Terminal care, Prognosis, Surveys and questionnaires

## Abstract

**Background:**

Depression is prevalent in people with very poor prognoses (days to weeks). Clinical practices and perceptions of palliative physicians towards depression care have not been characterised in this setting. The objective of this study was to characterise current palliative clinicians’ reported practices and perceptions in depression screening, assessment and management in the very poor prognosis setting.

**Methods:**

In this cross-sectional cohort study, 72 palliative physicians and 32 psychiatrists were recruited from Australian and New Zealand Society of Palliative Medicine and Royal Australian and New Zealand College of Psychiatrists between February and July 2020 using a 23-item anonymous online survey.

**Results:**

Only palliative physicians results were reported due to poor psychiatry representation. Palliative physicians perceived depression care in this setting to be complex and challenging. 40.0% reported screening for depression. All experienced uncertainty when assessing depression aetiology. Approaches to somatic symptom assessment varied. Physicians were generally less likely to intervene for depression than in the better prognosis setting. Most reported barriers to care included the perceived lack of rapidly effective therapeutic options (77.3%), concerns of patient burden and intolerance (71.2%), and the complexity in diagnostic differentiation (53.0%). 66.7% desired better collaboration between palliative care and psychiatry.

**Conclusions:**

Palliative physicians perceived depression care in patients with very poor prognoses to be complex and challenging. The lack of screening, variations in assessment approaches, and the reduced likelihood of intervening in comparison to the better prognosis setting necessitate better collaboration between palliative care and psychiatry in service delivery, training and research.

**Supplementary Information:**

The online version contains supplementary material available at 10.1186/s12904-022-00901-y.

## Background

Depression is a distressing condition for people with advanced life-limiting illnesses. It can reduce the quality-of-life of those affected and others around them, exacerbate physical suffering and worsen psycho-existential distresses [1–4]. Not only does depression impact patient engagement with their nearest supporters, but depression can also negatively affect clinicians’ ability to deliver care [1, 5]. Despite its prevalence, there is evidence that depression has been under-assessed and, even when recognised, under-managed in the palliative care setting [6–9].

In the palliative care population, there is a sub-group of patients with very poor prognoses defined as an estimated life expectancy in the range of days to weeks. This sub-group is characterised by a high degree of frailty, often with significant symptom burden and rapidly declining functional status [10, 11]. The frailty, symptom burden (e.g. fatigue, confusion, and dysphagia), and limited time for interventions to take effect can make depression assessment, psychotherapies and administration of typical antidepressants (e.g. Selective Serotonin Reuptake Inhibitors [SSRIs] and Serotonin Noradrenaline Reuptake Inhibitors [SNRIs]) challenging for clinicians [12–14]. Subsequently, clinicians’ approaches to depression assessment and management for these people might differ from palliative patients with better prognoses.

While previous studies of clinicians’ approaches to depression assessment and management in the general palliative care population have been done in Australia and the United Kingdom, palliative physicians’ and psychiatrists’ approaches to depression care specifically for people with very poor prognoses have not been explored [8, 9, 15].

## Methods

### Aim

This study aimed to characterise current Australasian palliative clinicians’ (palliative physicians and psychiatrists) reported practices and perceptions in depression assessment and management for palliative patients with very poor prognoses, including identifying barriers to optimising depression care in this context.

### Study Design

This was a cross-sectional cohort study using an online survey.

### Respondents

Eligible respondents were: 1. current members of the Australian and New Zealand Society of Palliative Medicine (ANZSPM), the largest Australasian professional society for medical practitioners interested in palliative medicine, including specialist physicians (e.g. palliative physicians and renal physicians), general practitioners, and radiation oncologists; and 2. psychiatry fellows and trainees registered with the Royal Australian and New Zealand College of Psychiatrists (RANZCP).

### Survey

The anonymous online survey (Additional file 1) used the Research Electronic Data Capture (REDCap) platform. It contained branching logic with a maximum of 23 questions (four multiple response questions and 19 single response questions) for each respondent, tailored according to the respondent’s self-identified primary discipline (palliative medicine or psychiatry) and previous encounters with patients with very poor prognoses. It explored the domains of depression screening, assessment, management and integration between psychiatry and palliative care services for patients with very poor prognoses based on extrapolation from the general palliative care literature and investigators’ clinical experiences [9, 14, 16, 17]. Particularly, interventions that might produce rapid antidepressant effects in the very poor prognosis setting such as adjunct antipsychotics, psychostimulants, ketamine and electroconvulsive therapy (ECT) were explored [18–21]. The survey contained two opened-ended questions asking for perceived challenges or barriers to effective assessment and management of depression in patients with very poor prognoses. To increase feasibility, validity and reliability, the survey questions were developed by the investigator panels consisting of clinical academic experts from palliative care and psychiatry and piloted with four palliative physicians without needing to further modify the questionnaire. The survey took, on average, 8 minutes to complete, on piloting.

### Recruitment

The survey link was first distributed by the professional bodies to members on the 25th of Feb 2020 (ANZSPM) and the 1st of May 2020 (RANZCP). Due to the restrictions of the survey dissemination policies, capacity for sending reminder emails was limited: for ANZSPM, only one reminder email was sent after 2 weeks; for RANZCP, no reminder email could be sent to the entire cohort but one reminder email was sent to the College Faculty of Consultation Liaison, after 6 weeks (12th of June 2020). Apart from the RANZCP mass cohort distribution, where the survey link was distributed as part of an electronic newsletter (Psyche), survey links were contained within the email distributed by the professional bodies (ANZSPM and RANZCP College Faculty of Consultation Liaison). The survey was closed on the 31st of Jul 2020. No financial incentives were offered to respondents.

### Data Analysis

Quantitative data were expressed as the number of respondents (percentage) and analysed using a IBM SPSS Statistics 26 [22].

Responses to the two open-ended items were analysed independently by two investigators (WL and MD) using conventional qualitative content analysis, which aligns with the aims of this study [23, 24]. WL was a palliative care physician who has clinical experience as a psychiatry resident, and MD was an experienced qualitative health researcher. Codes were developed inductively through careful reading of the data and sorted into categories of related material in NVivo 12. Categories were refined, defined, and subcategories developed through analyst discussion until consensus was achieved [23, 24]. Quantification of responses within subcategories was performed using NVivo 12 [25].

## Results

Completed surveys were obtained from 110 individuals: 79 responses out of 522 members of ANZSPM (15.1%); and 31 out of 6655 RANZCP members (0.5%). Of the 110 responses, 72 respondents identified as having the primary specialty of palliative medicine and 32 with psychiatry. Due to the lack of response from the RANZCP members and hence the lack of representation of the Australasian psychiatry cohort, only results from those who identified themselves primarily as palliative physicians (*n* = 72) were reported (Table 1).

**Table 1:** Demographics of Respondents

	Palliative Physicians (***n*** = 72) [n/%]
**Position**	
Specialist & Fellow	53 (73.6%)
Trainee	16 (22.2%)
Other	3 (4.2%)
**Training Background Apart From Palliative Medicine**	42 (58.3%)
GP	25 (34.7%)
Other Physician Training	13 (18.1%)
Critical Care (Emergency, Intensive Care, Anaesthetics)	1 (1.4%)
Psychiatry	1 (1.4%)
Other^a^	7 (9.7%)
**Gender**	
Male	18 (25.0%)
Female	54 (75.0%)
**Country**	
Australia	55 (76.4%)
New Zealand	17 (23.6%)
**Years Since Medical Graduation**	
< 10 years	8 (11.1%)
10–19 years	27 (37.5%)
20 or more years	37 (51.4%)
**Age**	
21–30	2 (2.8%)
31–40	20 (34.7%)
41–50	15 (20.8%)
51–60	23 (31.9%)
61–70	7 (9.7%)
71–80	0 (0.0%)
**Clinical Hours/ week**	
< 10	2 (2.8%)
10–19	5 (6.9%)
20–29	15 (20.8%)
30–39	32 (44.4%)
40 or more	18 (25.0%)
**Clinical Roles**^b^	
Community (patient home, group home and residential aged care facilities)	35 (48.6%)
Outpatient Clinic	35 (48.6%)
Consultative Service in Acute Hospital	45 (62.5%)
Acute Inpatient (Palliative Care or Psychiatry Wards in Acute Hospital)	28 (38.9%)
Subacute Hospital (Palliative Care Unit / Hospice / Subacute Psychiatry Unit)	30 (41.7%)
Encounter depression in very poor prognoses	70 (97.2%)^c^

^a^Other training backgrounds include Bioethics, Public Health, Pain Medicine, Oncology, Nursing, and General Paediatrics. ^b^Respondents could report multiple clinical roles. ^c^This number included a palliative medicine respondent (*n* = 1) who answered “Other” when asked about previous encounter of depression in the very poor prognosis setting due to difficulty in distinguishing pathological depressed mood from normal grief

Participating clinicians were mainly specialist and fellows (73.6%); female (75.0%); aged 31–60-year-old (87.4%); primarily working in Australia (76.4%); graduated more than 10 years ago (88.9%); and working ≥20 clinical hours per week (90.2%). Most clinicians (*n* = 70; 97.2%) reported having encountered depression in people with very poor prognoses.

The majority (*n* = 42; 58.3%) of all palliative physicians reported that they screen for depression in general palliative care patients, while only 40.0% (*n* = 28 out of 70) of clinicians encountering patients with very poor prognoses reported screening for depression.

Among physicians who might screen for depression (answered “yes” or “depends”) in general palliative care patients, the primary screening method reported was clinical interview (*n* = 53; 93.0%), followed by asking the family/carers (*n* = 40; 70.2%), asking other health professionals involved in the care (*n* = 37; 64.9%), and the use of screening tools (*n* = 27; 47.4%). For the very poor prognoses group, while 68.6% (*n* = 48) of physicians reported no difference in the way of screening compared to the general palliative population, 18.6% (*n* = 13) reported a “difference”: taking a more reactive rather than proactive approach; being briefer in assessment; relying more on objective information sources; and emphasising less on somatic symptoms. Among those who reported to use screening tools (n = 27), the most commonly used tools was the ultra-short two-items questionnaire (*n* = 14; 51.8%) followed by a single-item questionnaire (n = 5; 18.5%) (e.g. asking “Are you depressed” and/or “Have you had little interest or pleasure in doing things”). Only one respondent reported to use Hospital Anxiety and Depression Scale.

For depression assessment, at least 80% of physicians would ascertain whether the depression episode is first or recurrent during assessment, regardless of whether the prognoses is very poor or not. All physicians who have encountered depressed patients with very poor prognoses have experienced uncertainty regarding the cause of depression. Most palliative physicians (*n* = 56; 80.0%) would treat the depressed mood despite the uncertain cause. The primary sources of assistance sought by palliative physicians in this context were from psychiatry (*n* = 33; 47.1%) and psychology (*n* = 29; 41.4%).

For depression somatic symptom assessment, the majority (n = 37; 51.4%) of physicians reported including somatic symptoms in the general palliative care patients while excluding somatic symptoms in the sub-group with very poor prognoses (n = 29; 41.4%). Notably, in the setting of very poor prognoses, 30.0% (*n* = 21) of physicians reported “depends”: whether the somatic symptoms could be attributable to the nature of the terminal illnesses and associated interventions on an individual basis; and that somatic symptoms were still valuable to be considered in the “overall picture” of the patient.

For various treatment approaches for major depressive disorder in the setting of very poor prognoses (Table 2), most physicians reported using non-pharmacological approaches (*n* = 64; 91.4%), followed by the use of typical antidepressants (*n* = 63; 90%). When comparing the likelihood of using various depression interventions in the very poor prognoses sub-group as compared to the general palliative care cohort, the majority of physicians reported: no difference or less likely in using non-pharmacological interventions (both groups: *n* = 26; 37.1%), and less likely to use typical antidepressants (*n* = 36; 51.4%). For ECT in the setting of very poor prognosis, 72.9% (*n* = 51) and 14.3% (*n* = 10) of physicians reported to not use or less likely to use it respectively. There were bimodal distributions with the highest prevalence of “I don’t use” followed by “more likely to use” for treatment options of: atypical antipsychotics (n = 26; 37.1% - “I don’t use” and *n* = 20; 28.6% - “more likely”); benzodiazepines (n = 28; 40.0% - “I don’t use” and *n* = 24; 34.3% - “more likely”); and novel medication/experimental trials (*n* = 49; 70% - “I don’t use” and *n* = 12; 17.1% - “more likely”). Due to technical issues in the online survey platform, the psychostimulant item was initially not available for the first 28 participants, leading to the large proportion of non-response (n = 27; 38.6%) for this item. Despite this limitation, among the responders, the majorities answered “I don’t use” or “more likely to use” (both groups *n* = 18 out of 43; 41.9%).

**Table 2:** Clinicians’ Approaches to Major Depressive Disorder in People with Very Poor Prognoses Versus Better Prognoses

INTERVENTION	RESPONSE	PALLIATIVE PHYSICIANS *(n = 70)* *[counts (%)]*
a. Non-pharmacological interventions (e.g. supportive psychotherapy / counselling, cognitive therapy)	I don’t use	2 (2.9)
	Less likely (cumulative)	26 (37.1)
	No difference	26 (37.1)
	More likely (cumulative)	12 (17.1)
	No response	4 (5.7)
b. Typical antidepressant	I don’t use	3 (4.3)
	Less likely (cumulative)	36 (51.4)
	No difference	18 (25.7)
	More likely (cumulative)	9 (12.9)
	No response	4 (5.7)
c. Psychostimulant (e.g. methylphenidate, modafinil)^≠^	I don’t use	18 (25.7)
	Less likely (cumulative)	3 (4.3)
	No difference	4 (5.7)
	More likely (cumulative)	18 (25.7)
	No response	27 (38.6)
d. Atypical antipsychotics (e.g. risperidone, olanzapine)	I don’t use	26 (37.1)
	Less likely (cumulative)	6 (8.6)
	No difference	14 (20)
	More likely (cumulative)	20 (28.6)
	No response	4 (5.7)
e. Benzodiazepine	I don’t use	28 (40.0)
	Less likely (cumulative)	2 (2.9)
	No difference	12 (17.1)
	More likely (cumulative)	24 (34.3)
	No response	4 (5.7)
f. Novel medication / experimental trials (e.g. ketamine, esketamine nasal spray)	I don’t use	49 (70)
	Less likely (cumulative	4 (5.7)
	No difference	1 (1.4)
	More likely (cumulative)	12 (17.1)
	No response	4 (5.7)
g. Electroconvulsive therapy	I don’t use	51 (72.9)
	Less likely (cumulative)	10 (14.3)
	No difference	4 (5.7)
	More likely (cumulative)	1 (1.4)
	No response	4 (5.7)

≠ Due to a technical fault, the survey item exploring psychostimulant use was initially not accessible to the first 28 Australian and New Zealand Society of Palliative Medicine (ANZSPM) respondents

For service linkage with psychiatry (Table 3), the majority of palliative physicians reported to request for psychiatry input in an interval of monthly or longer (*n* = 41; 56.9%) and being requested by psychiatry for palliative care input yearly or longer (n = 26; 36.1%). Two-thirds of the palliative physicians (n = 48) thought contract frequency with psychiatry should be more frequent.

**Table 3:** Palliative Care and Psychiatry Service Linkage

***Palliative Physicians (n = 72)***		***Number (%)***
For assessment and management of depression in the overall palliative care setting, on average how often have you asked psychiatry for input?	Never	3 (4.2)
	Yearly or longer	16 (22.2)
	Monthly or longer	41 (56.9)
	Weekly or longer	6 (8.3)
	Daily or longer	0 (0.0)
	No response	6 (8.3)
For patients with depression and palliative care needs, on average how often have you been asked by psychiatry to provide palliative care management advice?	Never	24 (33.3)
	Yearly or longer	26 (36.1)
	Monthly or longer	15 (20.8)
	Weekly or longer	1 (1.4)
	Daily or longer	0 (0.0)
	No response	6 (8.3)
For optimal patient care, do you think contact frequency with psychiatry should be:	More frequent	48 (66.7)
	About right	9 (12.5)
	Other	9 (12.5)
	No response	6 (8.3)

Sixty-six respondents (91.7%) provided answers to the open-ended questions regarding key challenges or barriers to effective assessment and management of depression in palliative care patients with very poor prognoses. Respondents commented on the complexity of the clinical situation with interaction between physical, psychosocial, and spiritual dimensions. Reported key challenges and barriers are listed in Table 4, categorised under the domains of patient, clinician, health system, literature and society. On quantifying the various domain subcategories (Table 4), the three most frequently reported barriers were: the lack of therapeutic options that are rapidly effective (77.3%); the perceived frailty, burden and intolerance of depression assessment and management on the patient (71.2%); and the complexity in differentiating the symptoms of terminal illness from the somatic symptoms of depression (53.0%).

**Table 4:** Reported Challenges/Barriers to Depression Assessment and Management in People with Very Poor Prognoses

DOMAINS/SUBCATEGORIES	PREVALENCE OF REPORTING OF SUBCATEGORIES AMONG RESPONDENTS (***N*** = 66) (%)	EXAMPLE QUOTES
**Patient -** frailty, co-existing symptom burden and competing priorities of associated end-of-life issues when time for intervention effects is poor		
• Frailty, Burden & Intolerance*	71.2%	• “Fatigue, nausea, pain” (Participant 72) and “declining cognition” (Participant 27) • “Even when good psychology, psychiatry and/or pastoral care are available these patients are often too fatigued to participate in talking therapies” (Participant 25) • “Lack of effective medication which will make a difference without causing unnecessary side effects” (Participant 6)
• Therapeutic Efficacy - Lack of therapeutic options that are rapidly effective in the context of very poor prognoses*	77.3%	• “Time frame required for effect of pharmacologic and non pharmacologic interventions” (Participant 5) • “Timing and the poor prognosis which impedes any intervention to be effective.” (Participant 2)
• Competing priorities - Prioritisation of physical or other psychosocial & spiritual co-existing issues, symptoms or goals	21.2%	• “Competing priorities - physical symptoms and planning for end-of-life are often more pressing “(Participant 25) • “Other symptoms take priority and are focused on much more than mood disorders” (Participant 44)
**Clinician** - self-perceived limitations in psychiatry skills in the palliative care setting with incompetence in diagnostic differentiation		
Challenging diagnostic differentiation		
o Depression vs terminal illness symptoms*	53.0%	• “Challenges differentiating somatic symptoms from depression vs physical illness” (Participant 5) • “Usually hard to teese out how much is depression and how much is part of dying process” (Participant 13)
o Between depressed-mood syndromes or differentials (e.g. existential distress, demoralisation, adjustment disorder, organic brain syndrome)	19.7%	• “Challenges differentiating demoralisation from major depression” (Participant 5) • “Distinguishing between adjustment and depression” (Participant 8) • “Misattribution – e.g. depression with psychotic symptoms being attributed to delirium” (Participant 4)
o Normal vs Pathological	16.7%	• “Hard to distinguish from normal grief” (Participant 19) • “Difficulty assessing the difference between normal reactive mood changes [versus] pathological level of mood changes” (Participant 68)
• Limited Skills & Training	24.2%	• “Limited skills in psychiatric assessment - my last psychiatry placement was as a 3rd year medical student” (Participant 60) • “Limited knowledge of what works to improve mood in limited time frame” (Participant 41)
**System –** Inadequate health system resources and access to required interventions in the local health services		
• Suboptimal access and delivery of palliative care and mental health services	37.9%	• “High patient numbers for a small number of clinicians; Lack of allied health staff in [palliative care] MDT to deliver interventions” (Participant 31) • “Poor access to psychology/psychiatric services” (Participant 44)
• Lack of access to desired depression interventions	13.6%	• “Lack of access to resources for non-pharmacological management e.g. psychology, music therapy” (Participant 71) • “Access to rapid-acting medications like modafinil” (Participant 42)
• External Environment	1.5%	• “[Lack of] control of clinical envirmnment” (Participant 31)
• Language & Cultural issues	1.5%	• “Language / cultural barriers” (Participant 64)
**Literature** - Heterogeneity of depression concept and the lack of evidence to guide practice in the very poor prognosis setting		
• Lack of evidence & guidelines	15.2%	• “Uncertainty regarding the best treatment for this population/limited evidence base” (Participant 56)
• Heterogeneity of the concept and definition of depression in very poor prognosis setting	1.5%	• “Lack of defined criteria for diagnosis of depression in this group of patients” (Participant 48)
**Society –** Unsupportive attitudes and beliefs of patients, family and clinicians that prevents optimisation of depression care		
• Nihilism / Futility	10.6%	• “A sense of futility - Why assess it if there’s little I can do about it? “(Participant 25) • “Therapeutic nihilism” (Participant 21)
• Acceptance / Normalisation	12.1%	• “Acceptance that this [depression] is a normal part of end of life” (Participant 21) • “Normalisation” (Participant 40) • “Of course he/she is depressed, he/she is dying” (Participant 4)
• Resistance / Disinclination of patients, public, family or clinicians/staff	4.5%	• “Stigma” (Participant 65) • “Pressure from other health care professionals not to treat patients as they are dying” (Participant 34) • “Family not willing to engage non-pharm [interventions]” (Participant 64)

*Top three most commonly reported barriers: the lack of therapeutic options that are rapidly effective (77.3%); the perceived frailty, burden and intolerance of depression assessment and management on the patient (71.2%); and the complexity in differentiating the symptoms of terminal illness from the somatic symptoms of depression (53.0%)

## Discussion

This is the first study that captures palliative physicians’ practices and perceptions regarding depression care specifically in people with very poor prognoses of only days to weeks. As demonstrated by the survey, encountering depression in patients with very poor prognoses was common to palliative physician. However, despite the high prevalence of depression (up to 50%) in this population and the frequency of clinical encounters, only 40% of clinicians reported to screen for depression, with all clinicians reported to have experienced uncertainty when assessing the cause of depression [26]. This is reflected by the current study finding of the perceived challenging complexity of depression care in the very poor prognosis setting by clinicians. According to the literature, this complexity may be contributed to by the interplay of various domains of challenges reported in Table 4: 1) Patients’ frailty, co-existing symptom burden and associated end-of-life issues when time for intervention effects is poor [9, 14]; 2) Clinicians’ self-perceived limitations of psychiatry skills in the palliative care setting and incompetence in diagnostic differentiation [9, 27]; 3) Health system’s inadequacy of resources and access to required interventions in the local health services (e.g. mental health services) [8, 9]; 4) Heterogeneity of depression concept and the lack of evidence to guide practice in the literature for this context [26, 28]; and 5) Unsupportive societal attitudes that prevents the optimisation of depression care (e.g. stigma of mental illnesses, the “normalisation” or “acceptance” of depression at the end-of-life) [29, 30]. Each of these domains warrant future exploration for potential solutions to better optimise depression care in this setting.

Palliative physicians reported to less likely screen for depression and have ambivalence in depression assessment methods (e.g. approach to somatic symptoms of depression) in the very poor prognosis setting compared to the better prognosis setting. Diagnosing depression in the setting of very poor prognosis can be challenging as the symptoms of terminal illnesses (e.g. fatigue and weight loss) can confound the somatic symptoms of depression [17]. Importantly, this study shows that while clinicians may perceive somatic symptoms of depression to be less useful in depression diagnosis, somatic symptoms are still important to be considered during the overall depression assessment as they can affect the appropriateness of intervention choices. It may be desirable for clinicians to be trained with the various approaches to somatic symptoms such as Endicott Criteria to enable better diagnostic differentiation and depression assessment [31]. While they reported to generally intervene less in this setting (compared to patients with better prognoses), it is worth noting the bimodal distributions of clinicians not-using and more-likely-to-use certain non-typical pharmacological interventions (e.g. psychostimulants, atypical antipsychotics, benzodiazepines and novel medications such as ketamine) that have more augmentation and rapid-onset potentials than typical antidepressants [18–20, 32]. This may reflect clinicians’ attitudes where clinicians who were trained and aware of how to leverage the potential benefits of these non-typical treatments while minimising intolerance were more likely to embrace their use. Whereas, clinicians who lacked training or resources for these treatments did not tend to use them. Comparable to the study findings in the United Kingdom primary care and palliative settings, inadequately equipped clinicians may have a nihilistic attitude and ambivalence towards depression screening and assessment [8, 29, 30]. The low reported usage of ECT was likely related to clinicians perceiving the intervention to be over-burdensome for people with very poor prognoses [33]. Subsequently, palliative physicians and their multidisciplinary team members should be trained with the necessary skills to screen, assess, and administer first-line rapidly effective depression interventions in low-burden manners [34]. This may be facilitated by better linkage and integration of the psychiatry services into the palliative care services [17, 35].

Similar to the United States palliative physician cohort, near 70% of current survey’s respondents expressed desires for better collaboration with the psychiatry services [36]. At the clinical and health service levels, some strategies to improve palliative care and psychiatry collaboration might include: integrative multidisciplinary team [15, 36–38]; joint development of a tiered-referral model tailored to local health service needs [39]; and integrated clinician training via workshops and experiential training [35, 37, 38]. For research, palliative care and psychiatry researchers must collaborate to address barriers to the currently limited evidence base. On top of the barriers to depression care processes identified in this survey, other challenges include the effects of depression and terminal illnesses on participants’ ability to consent and engage with research activities, and the ethical concerns of trial participants receiving potentially ineffective therapies [40, 41]. There is a need for integrated palliative care and psychiatry research that explores appropriate depression screening and assessment strategies and potentially rapidly effective interventions using feasible and inclusive trial designs in the very poor prognosis setting (e.g. n-of-1, Bayesian response-adaptive-randomisation, or well-designed prospective case-controlled studies) [26, 35, 42]. Developing consensus approaches between palliative care and psychiatry via Delphi and updating the existing guideline based on the currently limited evidence to guide depression care specifically for people with very poor prognoses need to be considered [16, 17]. Overall, better collaboration between palliative care and psychiatry is urgently required, optimising timely access to needed interventions, complementing the shortfalls of both disciplines, and ultimately improving care to affected patients [35, 37, 43].

### Limitations

This study had low response rates, especially from the psychiatry cohort. These low rates were likely contributed by the COVID-19 pandemic, leading to clinicians focusing on COVID-19 related activities rather than non-COVID-19 research. It was possible that psychiatrists lacked interest or perceived a lack of relevance towards this topic due to their infrequent engagement with palliative care [35]. The low sample size limited the power for detailed subgroup analyses. The current survey did not include non-physician palliative clinicians (e.g. nurses and pastoral care) or psychologists. Furthermore, as the respondents were recruited only from the Australasia setting, the survey findings may not be generalised to non-Australasian contexts. Intrinsic to the study methodology, there was a risk of reporting bias where the reported practices deviate from the true practices. For depression interventions, various non-pharmacological interventions (e.g. supportive psychotherapy versus cognitive therapy) were not individually explored. Due to a technical fault, the survey question exploring psychostimulant use was initially unavailable to the first 28 ANZSPM respondents. Despite these limitations, the data collected still helped inform current practices and perceptions of some palliative physicians in Australasia. Lastly, while the prevalence data in Table 4 offered valuable insight into the prevailing perceived key barriers or challenges of depression care in the very poor prognosis setting among respondents, the prevalence data did not necessarily reflect the level of importance or influence of certain subcategories over another in optimising depression care. In fact, the domain subcategories reported less often such as the heterogeneity of depression concept and unsupportive societal attitudes might reflect that many clinicians were not cognisant of these topics, thus suggesting the need for improving awareness of these issues.

## Conclusions

Palliative physicians perceived depression care in people with very poor prognoses to be complex and challenging. The lack of screening, heterogeneity in the depression assessment, and the generally reduced likelihood of intervening for depression in the very poor prognosis setting compared to that of better prognosis highlighted the need for better collaboration between palliative medicine and psychiatry in health service delivery, clinician training, and research.

## Supplementary Information

**Additional file 1. **Current palliative care physicians’ and psychiatrists’ practices, challenges and potential improvement strategies in assessing and managing depression in palliative patients with very poor prognoses.

## Data Availability

The datasets used and analysed during the current study are available from the corresponding author on reasonable request.
