# Supplementary material for: Caring for depression in the dying is complex and challenging – survey of palliative physicians
Source: BMC Palliat Care. 2022 Jan 16;21:11. doi: 10.1186/s12904-022-00901-y (PMC8761382; doi:10.1186/s12904-022-00901-y)
Supplement: Supplementary file 1 — Additional file 1. Current palliative care physicians’ and psychiatrists’ practices, challenges and potential improvement strategies in assessing and managing depression in palliative patients with very poor prognoses. [file 12904_2022_901_MOESM1_ESM.pdf]

Current Palliative Care Physicians' and Psychiatrists' Practices, Challenges and Potential Improvement Strategies in Assessing and Managing Depression in Palliative Patients with Very Poor Prognoses

What is this project about?

Depression is a common and distressing condition for people facing advanced life limiting illnesses treated with palliative intent and their loved ones. Within this general palliative patient group, there is a sub-group of palliative patients with very poor prognosis, estimated to be measured in weeks at most. Their degree of frailty, poor functional status (mostly bedbound) and symptom burden can make depression assessment and management challenging. However, little is known about how depression is assessed and managed in this sub-group by palliative care physicians and psychiatrists, as well as their clinicians' perceived challenges and strategies to overcome these.

This project, therefore, aims to: 1. characterise the Australasian palliative care physicians' and psychiatrists' approaches to depression assessment and management in palliative care patients with very poor prognosis (estimated prognoses of days to weeks) compared to that of the other general palliative patients, and 2. find out their perceived challenges and potential improvement strategies.

Who is doing this project?

Dr Wei Lee, a palliative care physician at the Sacred Heart Hospital, Darlinghurst, Australia and PhD scholar at the University of Technology Sydney, is coordinating this project as part of his doctoral studies. This project is supervised by Prof David Currow & Prof Meera Agar (Professors of Palliative Medicine, University of Technology Sydney) and Prof Brian Draper (Professor of Psychiatry, University of New South Wales).

What does this project involve?

There are two parts to this study: Part A – an online survey; and Part B – an optional workshop / webinar. As an Australasian palliative care physician or psychiatrist, you are invited to participate. You have no obligation to participate if you don't feel inclined to.

The first part of this project is an online survey. There are, at maximum, 23 questions, which take on average 8 minutes to complete. This survey is completely anonymous.

At the end of the survey, you will also be invited to participate in a future one-hour workshop / webinar. This workshop may be set up at the location of the ANZSPM / RANZCP conference / meeting with webinar capacity so that no additional travel is required for you. It will aim to discuss the assessment and management of depression in palliative care patients with a very poor prognosis, including the survey results, integration between palliative care and psychiatry, perceived challenges and potential improvement. The discussion in this workshop will be recorded, transcribed and thematically analysed to produce the data required to create future recommendation.

Together, we can improve depression care at the end-of-life. Your consideration to participate in this project is greatly appreciated.

Survey Questions

DEMOGRAPHICS

1. Which of the following best represents you?
    a. Specialist/Fellow
    b. Trainee
    c. Other (Please specify)
2. What is your primary specialty?
    a. Palliative Medicine
    b. Psychiatry
    c. Others (Please specify)
3. Apart from your primary specialty, do you have another training background?
    a. General Practice
    b. Physician Training
    c. Psychiatry
    d. Critical care (Emergency, Intensive Care, Anaesthetics)
    e. Others

4. Which part of Australasia do you primarily work in?
    a. Australia
    b. New Zealand
    c. Other (Please Specify)

5. Years since medical graduation
    a. < 5
    b. 5-9
    c. 10-14
    d. 15-19
    e. 20 or more

6. Your age (year old)
    a. 21-30
    b. 31-40
    c. 41-50
    d. 51-60
    e. 61-70
    f. 71-80
    g. > 81

7. Your gender:
    a. Male
    b. Female
    c. Other
    d. Rather not say

8. On the usual basis, how many hours per week do you take on the role as a clinician on average?
    a. <10

b. 10-19
c. 20-29
d. 30-39
e. 40 or more

9. Where do you spend time in your clinical role (Tick all that apply)?
   a. Community setting (Patient's home, group home and / or residential aged care facilities)
   b. Outpatient Clinic
   c. Consultative service in acute hospitals
   d. Acute inpatient care (palliative care or psychiatry wards in acute hospital)
   e. Subacute hospital (e.g. palliative care unit / hospice, subacute psychiatry unit)

ENCOUNTER

10. Have you encountered depression in palliative care patients with very poor prognoses (defined in this project as those with estimated prognoses in the range of days to weeks, characterised by them being mostly bedbound)?
    a. Yes → (Continue survey)
    b. No → (Jump questions containing palliative care patients with very poor prognoses using branching logic electronically)
    c. Other (Please – Specify)

ASSESSMENT

11. Do you screen for depression in palliative care patients?
    a. Yes → continue
    b. No → jump to Q15
    c. Depends (Please – Specify)

12. How do you screen for depression in these patients? (Tick all that apply)
    a. By seeing / interviewing the patient
    b. By using a tool (Please select / name the tools – drop down menu: Hospital Anxiety and Depression Scale, asking one item question: "Are you depressed?", asking two items "Are you depressed", "Have you had little interest or pleasure in doing things" (e.g. Patient Health Questionnaire-2), Geriatric Depression Scale, Beck's Depression Inventory, Others [Please-Specify])
    c. By asking the family / carers
    d. By asking other health professionals involved in the care (e.g. nurses, social worker, clinical psychologist, pastoral care worker)
    e. Other (free-texts)

13. For the subgroup of palliative care patients with very poor prognoses, do you screen?
    a. Yes → continue
    b. No → jump to Q15
    c. Depends (Please – Specify)

14. Is your method of screening for depression in palliative care patients with very poor prognoses different to your method of screening in other palliative care patients?
    a. No different
    b. Different (Please specify the differences using free text)
    c. Depends (Please – specify)

15. Do you exclude somatic symptoms of depression (e.g. changes in weight, sleep, fatigue and inability to concentrate) when assessing for depression?
    a. In palliative care patients without very poor prognoses (Drop down list: Exclude / Include / Depends (Please – Specify)
    b. In palliative care patients with very poor prognoses (Drop down list: Exclude / Include / Depends (Please – Specify)

16. What do you think are the key challenges/ barriers to effective **assessment** of depression in palliative care patients with very poor prognoses? (List up to 3 with free-text)
    a.
    b.
    c.

<u>MANAGEMENT</u>

17.
I. (Palliative Care Physician only) What would you usually do when you encounter palliative care patients with very poor prognoses with debilitating depression that affects their functional ability but you are uncertain of the cause (Tick all that apply)?
    a. Treat the depressed mood (using non-pharmacological and/or pharmacological interventions)
    b. Request for a second opinion from other palliative care colleagues
    c. Request for psychology input
    d. Request for psychiatry input
    e. Other (Please specify)
    f. That has not happened to me

II. (Psychiatry Physician only) What would you usually do when you encounter palliative care patients with very poor prognoses with debilitating depression that affects their functional ability but you are uncertain of the cause (Tick all that applies)?
    a. Treat the depressed mood (using non-pharmacological and/or pharmacological interventions)
    b. Request for a second opinion from other psychiatric colleagues
    c. Request for psychology input
    d. Other (Please specify)
    e. That has not happened to me

18. Do you usually ascertain whether the depression experienced by the patient is the first or recurrent episode?

a. In palliative care patients without very poor prognoses (Drop down menu: Yes / No /Depends (Please specify)
b. In palliative care patients with very poor prognoses (Drop down menu: Yes / No /Depends (Please specify)

19. Comparing the treatment of major depressive disorder in palliative care patients with very poor prognoses to treating that in the other palliative care patients, how likely are you going to use each of the following treatment during your routine practice?
0 = I don't use this treatment for treating major depressive disorder
1 = Much less likely than in the other palliative care patients
2 = Less likely than in the other palliative care patients
3 = No difference to the treatment in the other palliative care patients
4 = More likely than in the other palliative patients
5 = Much more likely than in the other palliative patients

a. Non-pharmacological interventions (e.g. supportive psychotherapy / counselling, cognitive therapy)	0, 1, 2, 3, 4, 5
b. Typical antidepressant	0, 1, 2, 3, 4, 5
c. Psychostimulant (e.g. methylphenidate, modafinil)	0, 1, 2, 3, 4, 5
d. Atypical antipsychotics (e.g. risperidone, olanzapine)	0, 1, 2, 3, 4, 5
e. Benzodiazepine	0, 1, 2, 3, 4, 5
f. Novel medication / experimental trials (e.g. ketamine, esketamine nasal spray)	0, 1, 2, 3, 4, 5
g. Electroconvulsive therapy	0, 1, 2, 3, 4, 5

20. What do you think are key challenges/ barriers to effective **management** of depression in palliative care patients with very poor prognoses? (List up to 3 using free-text)
    a.
    b.
    c.

PSYCHIATRY LINKAGE

For palliative care physicians only:

21. For assessment and management of depression in the overall palliative care setting, on average how often have you asked psychiatry for input? (1= Never; 2 = yearly or longer; monthly or longer; 4 weekly or longer 5 daily or longer)
22. For patients with depression and palliative care needs, on average how often have you been asked by psychiatry to provide palliative care management advice? (1= Never; 2 = yearly or longer; monthly or longer; 4 weekly or longer 5 daily or longer)
23. For optimal patient care, do you think contact frequency with psychiatry should be: (more frequent; less frequent; about right; others)

For psychiatrists only:

21. For patients with depression and palliative care needs, on average how often have you ask palliative care for management input? (1= Never; 2 = yearly or longer; monthly or longer; 4 weekly or longer 5 daily or longer)
22. For assessment and management of depression in the overall palliative care setting, on average how often would you get asked by palliative care for input? (1= Never; 2 = yearly or longer; monthly or longer; 4 weekly or longer 5 daily or longer)
23. For optimal patient care, do you think contact frequency with palliative care should be: (more frequent; less frequent; about right; others)

Thank you very much for participating in this survey. This is the end of the survey.

If you are willing participate in the subsequent one-hour workshop / webinar (alongside other surveyed clinicians in the same discipline) to learn about survey results, discuss more about issues and challenges in this setting and offer potential strategies for improvement, please kindly leave your contact detail using a separate online link attached in this email. This will help us organise this workshop/ webinar.

(A separate online link in this email is used for registering your contact details to ensure that the anonymity of this survey is maintained – i.e. your *personal information will not be attached to your survey results).*
